# Large-scale expansion of Wharton’s jelly-derived mesenchymal stem cells on gelatin microbeads, with retention of self-renewal and multipotency characteristics and the capacity for enhancing skin wound healing

**DOI:** 10.1186/s13287-015-0031-3

**Published:** 2015-03-19

**Authors:** Guifang Zhao, Feilin Liu, Shaowei Lan, Pengdong Li, Li Wang, Junna Kou, Xiaojuan Qi, Ruirui Fan, Deshun Hao, Chunling Wu, Tingting Bai, Yulin Li, Jin Yu Liu

**Affiliations:** Department of Pathobiology, Key Laboratory of Ministry of Education, College of Basic Medicine, Jilin University, Changchun, 130021 P.R. China; Department of Toxicology, School of Public Health, Jilin University, Changchun, 130021 P.R. China; Harbin Veterinary Research Institute, CAAS – Michigan State University Joint Laboratory of Innate Immunity, State Key Laboratory of Veterinary Biotechnology, Chinese Academy of Agricultural Sciences, Harbin, Heilongjiang 150001 P R China

## Abstract

**Introduction:**

Successful stem cell therapy relies on large-scale generation of stem cells and their maintenance in a proliferative multipotent state. This study aimed to establish a three-dimension culture system for large-scale generation of hWJ-MSC and investigated the self-renewal activity, genomic stability and multi-lineage differentiation potential of such hWJ-MSC in enhancing skin wound healing.

**Methods:**

hWJ-MSC were seeded on gelatin microbeads and cultured in spinning bottles (3D). Cell proliferation, karyotype analysis, surface marker expression, multipotent differentiation (adipogenic, chondrogenic, and osteogenic potentials), and expression of core transcription factors (OCT4, SOX2, NANOG, and C-MYC), as well as their efficacy in accelerating skin wound healing, were investigated and compared with those of hWJ-MSC derived from plate cultres (2D), using in vivo and in vitro experiments.

**Results:**

hWJ-MSC attached to and proliferated on gelatin microbeads in 3D cultures reaching a maximum of 1.1–1.30 × 10^7^cells on 0.5 g of microbeads by days 8–14; in contrast, hWJ-MSC derived from 2D cultures reached a maximum of 6.5 -11.5 × 10^5^ cells per well in a 24-well plate by days 6–10. hWJ-MSC derived by 3D culture incorporated significantly more EdU (*P* < 0.05) and had a significantly higher proliferation index (*P* < 0.05) than those derived from 2D culture. Immunofluorescence staining, real-time PCR, flow cytometry analysis, and multipotency assays showed that hWJ-MSC derived from 3D culture retained MSC surface markers and multipotency potential similar to 2D culture-derived cells. 3D culture-derived hWJ-MSC also retained the expression of core transcription factors at levels comparable to their 2D culture counterparts. Direct injection of hWJ-MSC derived from 3D or 2D cultures into animals exhibited similar efficacy in enhancing skin wound healing.

**Conclusions:**

Thus, hWJ-MSC can be expanded markedly in gelatin microbeads, while retaining MSC surface marker expression, multipotent differential potential, and expression of core transcription factors. These cells also efficiently enhanced skin wound healing in vivo, in a manner comparable to that of hWJ-MSC obtained from 2D culture.

## Introduction

Mesenchymal stem cells (MSCs), originating from the mesoderm, have a capacity for self-renewal and potential for multilineage differentiation into adipocytes, smooth muscle cells, chondrocytes, and osteoblasts [1-4]. An increasing number of studies have shown that MSCs play unique roles in routine physiological activities and pathological responses, including tissue homeostasis, aging, immune regulation, inflammatory response, apoptosis, and tissue repair and regeneration [5-7]. Moreover, increasingly, there is evidence that MSCs can be derived from multiple tissues and organs, including bone marrow [8], umbilical cord [9], fat [10], placenta [11], dermis [12], hair follicles [13], and body fluids, such as amniotic fluid [14], umbilical cord blood [15], peripheral blood [16], urine [17], and menstrual blood [18]. Among these tissues and organs, Wharton’s jelly from the human umbilical cord is readily available, easily harvested, and is a rich source of human Wharton’s jelly-derived mesenchymal stem cells (hWJ-MSC). hWJ-MSC exhibit marked advantages over MSCs derived from other sources. Given their low immunogenicity and the lack of associated ethical concerns [19-21], hWJ-MSC can be viewed as an attractive cell source for cell-based tissue engineering [22], gene therapy [23], and cell therapy [24].

Successful stem cell therapy requires billions of stem cells [25,26]. Such vast numbers of stem cells are traditionally achieved by repeated subcultures of stem cells in tissue culture plates or flasks (2D cultures), but this inevitably leads to cell senescence and loss of multipotency, compromising stem cell therapy in regenerative medicine. There is, therefore, a need for the development of a novel technique that yields stem cells on a large scale while maintaining the high proliferation potential and multipotency of the cells.

Spinning bottle cell culture systems (3D cultures) offer advantages in terms of optimal use of culture space, as well as the automatic and efficient optimization of culture conditions [27,28], such as discharge of waste products, replacement of medium, and maintenance of pH. Various 3D cell culture systems have been developed, including perfusion cell culture systems [29], rotary cell culture systems [30], and stirred suspension cell culture systems [31]. The successful development of a 3D culture system for large scale generation of stem cells mitigates the abovementioned shortcomings related to expansion of stem cells in tissue culture plates [32].

Among the 3D culture systems mentioned above, the stirred suspension culture system, which utilizes microbeads as microcarriers for cell culture, has been considered the most efficient approach for expanding MSCs on a large scale, and was first introduced by Van Wezel *et al*. in mass production of vaccines [33]. Recently, the microcarrier cell culture system has been used for expansion of embryonic stem cells [27], bone marrow-derived MSCs [34], skin-derived keratinocytes [35], melanocytes, and fibroblasts [36,37], and induced pluripotent stem cells [38]. Moreover, stem cells produced in a microcarrier cell culture system retain their multipotency [38]. Promisingly, studies have shown that keratinocytes [39], which had been highly expanded on gelatin microbeads, can be delivered directly to wounded or diseased skin sites in order to treat recalcitrant leg ulcers and stable vitiligo with great success.

In this study, we aimed to generate hWJ-MSC on a large scale by using macroporous gelatin microbeads as microcarriers for cell culture and spinning bottles as culture vessels, to explore the proliferative capacity, multipotency, stemness, and biological efficacy of these cells in promoting skin wound healing.

## Methods

### Isolation of hWJ-MSC

This study was approved by the Ethics Committee of Basic College of Medicine, Jilin University (China). The umbilical cords were obtained from healthy patients who granted prior informed written consent in the First Hospital of Jilin University. Isolation of hWJ-MSC has been described elsewhere [40,41]. Briefly, umbilical cords were washed extensively in phosphate-buffered saline (PBS, Dingguo Biotech, Beijing, China) containing 100 μg/mL streptomycin and 100 U/mL penicillin (Hyclone, Victoria, Australia). Wharton’s jelly tissues were then stripped from the middle of the umbilical cords using forceps, cut into small pieces with scissors, and digested in 0.1% collagenase type I (Invitrogen, Carlsbad, CA, USA) for 16 hours at 37°C to release hWJ-MSCs. hWJ-MSC were collected by centrifugation at 402 g for five minutes and cultured in (Dulbecco’s) modified Eagle’s medium/F12 ((D)MEM/F12, Invitrogen) containing 10% fetal bovine serum (FBS; Hyclone, Victoria, Australia) and 10 ng/mL basic fibroblast growth factor (bFGF; Peprotech, London, UK) in tissue culture plates, at 37°C under 5% CO_2_. The medium was changed every three days. When the hWJ-MSC had proliferated to 80% confluence, they were subcultured and used as cell sources in the subsequent experiments.

Phenotypic characterization and the trilineage differentiation potential of hWJ-MSC (toward adipocytes, chondrocytes, and osteoblasts) were assayed (see the Immunofluorescence staining and flow cytometry assay and the Multipotency assay subsections).

### Microcarrier preparation and establishment of a 3D culture system

The establishment of a 3D culture system has been described elsewhere [13]. Briefly, 0.5 g of macroporous gelatin microbeads (CultiSpher-G, Percell Biolytica AB, Sigma–Aldrich, St. Louis, MO, USA) were washed and rehydrated in 50 mL PBS overnight at 4°C. After autoclaving, the microcarriers were washed with PBS followed by culture medium and were then transferred into spinning bottles (Cellspin, Chur, Switzerland). According to product specifications, 0.5 g of these microbeads represents a surface area of 0.5 m^2^, and a well in a 24-well plate has a surface area of 2 cm^2^. We seeded 0.5 × 10^6^, 2.5 × 10^6^, and 5 × 10^6^ hWJ-MSCs on 0.5 g gelatin microbeads and 200, 1,000, and 2,000 hWJ-MSC in wells of 24-well plates, to achieve a final seeding density of 100 cells/cm^2^, 500 cells/cm^2^, and 1,000 cells/cm^2^ on microbeads or in 24-well plates, respectively. hWJ-MSCs cultured on microbeads were designated as 3D cultures and those grown in 24-well plates as 2D cultures. The hWJ-MSC–microbeads mixtures were cultured in spinning bottles, and the stirring mode was set to 20 minutes stationary and 40 minutes spinning at 25 rpm. When the hWJ-MSC had attached to CultiSpher-G completely (as evidenced by acridine orange dye staining), the stirring mode was set to 5 minutes stationary and 55 minutes spinning at 50 rpm. Culture medium was changed every three days. All the cells were cultured under 5% CO_2_ at 37°C and the medium used for the 3D cultures was the same as that used for the 2D cultures.

### Acridine orange dye staining and cell proliferation assay

hWJ-MSC (triplicate samples) in 2D or 3D cultures were released from tissue culture plates and microbeads by trypsinization and enumerated using a hemocytometer, every other day. Cell proliferation curves were created by plotting the numbers of cells counted over the culture periods (days). Cells were stained with acridine orange as described previously [42]. Briefly, cell suspensions were incubated with 200 μL of 100 μg/mL acridine orange dye for 20 minutes at 37°C.

### Cell cycle assay

A cell cycle assay was performed as described previously [43]. Briefly, hWJ-MSC were released from triplicate 2D or 3D cultures by trypsinization, washed in cold PBS, and fixed in 70% cold ethanol (4°C, 12 hours). After fixation, the cells were washed in cold PBS and stained with 50 μg/mL propidium iodide staining solution containing 200 mg/mL RNase A (Invitrogen) at 37°C for 30 minutes in the dark. After staining, the cell cycle of hWJ-MSC was analyzed by flow cytometry (BD Biosciences, Franklin Lakes, NJ, USA) and the cell proliferation index (PI) was calculated using the equation

$$ \left(\mathrm{PI}\right) = \left(\mathrm{S} + \mathrm{G}2\mathrm{M}\right)/\left(\mathrm{G}0/1 + \mathrm{S} + \mathrm{G}2\mathrm{M}\right) $$

### Assessment of senescence-associated β-galactosidase staining

Passage 6 hWJ-MSC from 2D and 3D cultures were seeded into six-well plates, with 1 × 10^5^ cells/well. After 24 hours culturing, the cells were stained for senescence-associated β-galactosidase (SA-β-gal) to detect cell senescence, using the Senescence-Associated β-Galactosidase kit (Beyotime, Beijing, China) according to the instructions of the manufacturer. Senescent cells were observed using an optical microscope and counted in three random fields of vision (200 cells per field were used to calculate level of positivity) [44].

### Karyotype analysis

Karyotype analysis of passage 6 hWJ-MSC from 2D and 3D cultures was performed at the First Bethune Hospital of Jilin University, using standard protocols for high-resolution G-banding.

### 5-Ethynyl-2′-deoxyuridine incorporation

5-Ethynyl-2′-deoxyuridine (EdU) is an analogue of thymidine, which is incorporated into dividing cells during DNA synthesis. Therefore, EdU incorporation is an indicator of cell proliferation. An EdU incorporation assay was performed with an EdU detection kit, as suggested by the manufacturer (Ribobio, Guangzhou, China). Briefly, hWJ-MSCs in the exponential phase in 2D (day 6) or 3D (day 6) cultures were incubated in medium containing a final concentration of 50 μM EdU for 12 hours at 37°C under 5% CO_2_. Subsequently, hWJ-MSC were fixed with 4% paraformaldehyde (room temperature, 30 minutes; Dingguo, Beijing, China) and permeabilized with 0.5% TritonX-100 (Sigma–Aldrich) for five minutes. hWJ-MSC were then washed with PBS and stained with 1 × Apollo® staining solution at room temperature for 30 minutes, followed by counterstaining with Hoechst 33342 dye (room temperature for 10 minutes; Invitrogen). hWJ-MSC were observed using a fluorescence microscope (4000B, Leica Microsystems, Wetzlar, Germany) and photographed using a digital camera (Leica, DFC500, Wetzlar, Germany) fitted to the microscope. The numbers of EdU-labeled hWJ-MSC were counted in five randomly selected views and normalized to the nucleus.

### Immunofluorescence staining and flow cytometry assay

The expression of mesenchymal stem cell surface markers and embryonic stem cell (ESC) markers was inspected using immune fluorescence, as describe previously [41]. Briefly, hWJ-MSC were released from 2D and 3D cultures and incubated with primary mouse anti-human antibodies against CD31, CD44, CD45, CD73, CD90, and CD105 (BD Biosciences, Franklin Lakes, NJ, USA), or rabbit anti-human antibodies against SOX2, NANOG, and C-MYC (Cell Signaling, Beverly, MA, USA) and OCT4 (Abcam, Cambridge, UK) at a dilution of 1:200 at 4°C overnight. After three washes in PBS, hWJ-MSC were incubated with Alexa Fluor 488-conjugated anti-rabbit/mouse (Cell Signalling) or Alexa Fluor 647-R-phycoerythrin-conjugated rabbit anti-mouse secondary antibodies (1:300 dilution; Cell Signalling) at room temperature for 60 minutes in the dark. hWJ-MSC were then washed three times in PBS and counterstained with Hoechst 33342 (1:10,000 dilution; Invitrogen) at room temperature for five minutes in the dark. Subsequently, cells were observed using a fluorescence microscope (Leica, DMI4000B) and photographed using a digital camera (Leica, DFC500).

For flow cytometry assays, cells were treated with the same procedures as for immunofluorescence staining, and hWJ-MSC were then analyzed using a FACS Calibur flow cytometer (BD Biosciences, San Jose, CA, USA). Data were analyzed using Cell Quest Software (BD Biosciences).

### Multipotency assay

The adipogenic and osteogenic differentiation of hWJ-MSCs was performed as described previously [41]. Briefly, the cells were cultured in either adipogenic medium, consisting of (D)MEM, 10% FBS, 1 μM dexamethasone (Sigma–Aldrich), 0.5 mM isobuty1-methylxanthine (Sigma–Aldrich), 200 μM indomethacin (Sigma–Aldrich), and 10 μM insulin (Sigma–Aldrich), or in osteogenic medium, consisting of (D)MEM, 10% FBS, 10 mM β-glycerophosphate (Alfa Aesar, Ward Hill, MA, USA), 0.1 μM dexamethasone, and 50 μM ascorbate-2-phosphate (Sigma–Aldrich). The medium was changed every three days. Three weeks after adipogenic and osteogenic induction, intracellular lipid droplets were subjected to oil red O staining and mineralized bone nodules were detected using Alizarin Red-S staining [45].

After staining, isopropanol (Beijing Chemical Works, Beijing, China) was added to the cells to dissolve the oil red O accumulated in the hWJ-MSC. The optical density (OD) of the oil red O was measured using a microplate reader (Tecan, Infinite 200 PRO, Grödig, Austria) at a wavelength of 490 nm and 10% cetylpyridinium chloride (Sigma–Aldrich) was added to dissolve the accumulated alizarin red S, and the OD was measured using a microplate reader at a wavelength of 560 nm.

For chondrogenic differentiation, hWJ-MSC spheres [41] were generated by hanging drop culture, utilizing 20 μL of 8 × 10^6^ cells/mL; spheres were cultured in chondrogenic-induction medium consisting of (D)MEM, 10% FBS, 6.25 μg/mL insulin, 10 ng/mL transforming growth factor-beta 1 (PeproTech, London, UK), and 50 nM of ascorbate-2-phosphate (Sigma–Aldrich). The culture medium was replaced every three days. Three weeks after chondrogenic induction, cartilage were detected with toluidine blue (Dingguo, Beijing, China) staining according to the specification of the manufacturer [45].

### Reverse transcription and real time PCR

Total RNA was extracted from adipocytes, osteoblasts, and chondrocytes derived from hWJ-MSC in 2D and 3D cultures using Trizol (Invitrogen) according to the manufacturer’s instructions, and reverse transcribed into cDNA using a Reverse Transcriptase kit (TRANSGEN, Beijing, China) as suggested by the manufacturer. The cDNA (2 μL) was then used as the template for PCR amplification. Real-time PCR was performed with SYBR Green PCR Master Mix (Roche Diagnostics, Basel, Switzerland) on a Real-Time PCR System (ABI Prism 7300 Sequence Detection System, Applied Biosystems, Forster City, CA, USA) [46]. The fold-changes in cDNA levels of the target gene, after normalization to levels of *GAPDH* as a reference gene, were determined using the following formula: fold-change 2^-ΔΔCt^, where ΔΔCt = (Ct_Target_ − Ct_GAPDH_) for the sample − (Ct_Target_ − Ct_GAPDH_) for the control. The primers used for PCR and real-time PCR are shown in Table 1.

**Table 1:** **List of primers used for PCR and for real-time PCR gene expression analyses**

**Name**	**Primer**	**Sequence**	**Product size**	**GenBank Accession number**
*RUNX2*	Forward	5′-TGGTTAATCCGCAGGTCAC-3′	70 bp	NM_001015051.3
	Reverse	5′-ACTGTGCTGAAGAGGCTGTTTG-3′		
*AP*	Forward	5′-AAAGAAGTAGGAGTGGGCTTTGC-3′	79 bp	NM_001442.2
	Reverse	5′-CCCCATTCACACTGATGATCAT-3′		
*Collagen II*	Forward	5′-AGAGACCTGAACTGGGCAGA-3′	215 bp	NM_006719242.1
	Reverse	5′-TGACACGGAGTAGCACCATC-3′		
*SOX2*	Forward	5′-TTGCTGCCTCTTTAAGACTAGGA-3′	75 bp	NM_003106.3
	Reverse	5′-CTGGGGTAAACTTCTCTC-3′		
*C-MYC*	Forward	5′-CAGCTGCTTAGACGCTGGATTT-3′	115 bp	NM_002467.4
	Reverse	5′-ACCGAGTCGTAGTCGAGGTCAT-3′		
*NANOG*	Forward	5′-CCAACATCCTGAACCTCAGCTAC-3′	121 bp	NM_024865.2
	Reverse	5′-GCCTTCTGCACACCATT-3′		
*OCT4*	Forward	5′-CAAAGCAGAAACCCTCGTGC-3′	171 bp	NM_001285987.1
	Reverse	5′-AACCACACTCGGACCACATC-3′		
*GAPDH*	Forward	5′-ACATCAAGAAGGTGGTGAAGCAGG-3′	123 bp	XM_005253678.1
	Reverse	5′-TATCGTCAAAGGTGGAGGAGTGG-3′		

### Protein extraction and western blot analysis

The same number of hWJ-MSC obtained via 2D or 3D cultures were lysed in ice-cold cell radioimmunoprecipitation assay (RIPA) buffer (Beyotime, Shanghai, China) for 30 minutes on ice and were then centrifuged at 16,000 × *g* for 10 minutes at 4°C. The supernatant was collected and the total protein concentration was determined using a BCA kit (Beyotime). An amount of 20 μg total protein was fractionated by 10% SDS-PAGE and electroblotted onto 0.22-μm polyvinylidene fluoride (PVDF) membranes (Beyotime). Membranes were then blocked with 5% skim milk in TBST (10 mM Tri-HCL, 150 mM NaCl, 0.25% Tween-20, pH 7.5) at room temperature for one hour, followed by overnight incubation with the following primary antibodies: polyclonal rabbit anti-human SOX2, C-MYC, NANOG (all 1:100 dilution, Cell Signalling), and goat-anti-human OCT4 (1:100 dilution, Abcam). Mouse-anti-human β-actin (1:100 dilution; Abcam) served as the protein-loading control. After washing with TBST, the membranes were incubated for one hour at room temperature with goat anti-rabbit immunoglobulin G-horseradish peroxidase (IgG-HRP) secondary antibody (Santa Cruz, Dallas, TX, USA, 1:1000 dilution) in the case of SOX2, C-MYC, and NANOG; with goat anti-mouse IgG-HRP (1:1000 dilution) for detection of β-actin; or with mouse anti-goat IgG-HRP (1:1000 dilution) for OCT4. After three washes with TBST, proteins were visualized using GENE GNOME (Gene Company Ltd, Hong Kong, China). The relative amount of proteins on the blots was determined using a Gel Image System (Tanon, Shanghai, China).

### Cell transplantation and skin wound healing

All animal procedures were conducted in compliance with the guidelines approved by the China Association of Laboratory Animal Care and the Institutional Animal Care Committee. Balb/C mice (male, 20 to 25 g,) were purchased from Fukang Animal Breading Center, Beijing, China, and kept at the Institutional Animal Center, Jilin University, China. Mice were acclimated to their environment for one week, after which a 0.8 cm × 0.8 cm square, full-thickness excisional wound was created on the dorsal skin of each mouse using surgical scissors. Immediately thereafter, 100 μl PBS (PBS group, n = 6), 1 × 10^6^ hWJ-MSC derived from 2D culture (2D group, n = 6) or from 3D culture (3D group, n = 6), in 100 μl of PBS, was injected into the dermis at the four corners of the wound (25 μl per corner). Then, a single layer of oil gauze was used as the primary wound dressing; this was covered by three layers of cotton gauze. The sham group consisted of six mice that had received neither PBS nor hWJ-MSC injections.

At days 3, 7, 14, and 21 after cell implantation, photographs were taken of the wound area for gross inspection of wound closure. The wound outline was depicted along the wound margin using transparent film, and wound closure was calculated as follows: (original wound area – new wound area)/original wound area × 100%. Mice were then sacrificed and skin samples, including the wound and neighboring tissues, were taken for histological inspection. The skin samples were fixed with 10% buffered formaldehyde, embedded in paraffin, sectioned at 6 μm, and stained with hematoxylin and eosin (H & E). Slices were observed using a microscope and photographed. The wound area was measured by tracing the open section of the epidermis under the microscope (Olympus, Tokyo, Japan) using Image J software (National Institutes of Health). Epidermal tissue lacking hair follicles that was present on the dermis was defined as newly generated epidermis. Histological wound healing was calculated as follows: length of newly generated epidermis (length of newly generated epidermis + length of wound) × 100%.

Immunofluorescent staining was performed to detect epidermal genesis and angiogenesis in the skin samples. Briefly, after deparaffinization and rehydration, skin slides were incubated with 1% BSA/PBS at room temperature for 30 minutes to block nonspecific binding. Next, the slides were incubated with rat anti-mouse primary antibody against CD31 or cytokeratin 14 (1:200 dilution; both Abcam) at 4°C overnight. The slides were then washed three times in PBS, and incubated with Alexa Fluor-488/555-conjugated anti-mouse secondary antibody at room temperature for 30 minutes. The slides were then counterstained with Hoechst 33342 to track the nucleus, and viewed and photographed under a microscope.

### Statistical analysis

Statistical analysis was performed using SPSS 17.0 (SPSS Inc., Chicago, IL, USA). Data are presented as the means ± standard deviation (SD) from at least three independent experiments. Multiple group comparisons were made using one-way analysis of variance (ANOVA). Two group comparisons were made using two-way ANOVA with *post hoc* tests. *P* <0.05 was considered statistically significant.

## Results

### Isolation of hWJ-MSC

**Figure 1:**
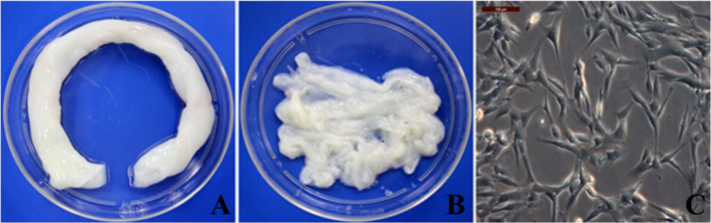
**Isolation of hWJ-MSC.** Human umbilical cords were collected from full-term deliveries **(A)**, and Wharton’s jelly was harvested. **(B)**, Bar = 100 μm. hWJ-MSC were released from Wharton’s jelly tissue by digestion of the umbilical cord with collagenase; cells exhibited an elongated and polygon-like morphology in tissue culture plates **(C)**. hWJ-MSC, human Wharton’s jelly-derived mesenchymal stem cells.

Human umbilical cords were collected in cases of full-term delivery (Figure 1A). Wharton’s jelly tissues were removed from umbilical cords (Figure 1B) and digested with collagenase to release mesenchymal stem cells (hWJ-MSC). hWJ-MSCs exhibited elongated and polygon-like morphology in tissue culture plates (Figure 1C).

### Proliferation of hWJ-MSC in 2D and 3D cultures

**Figure 2:**
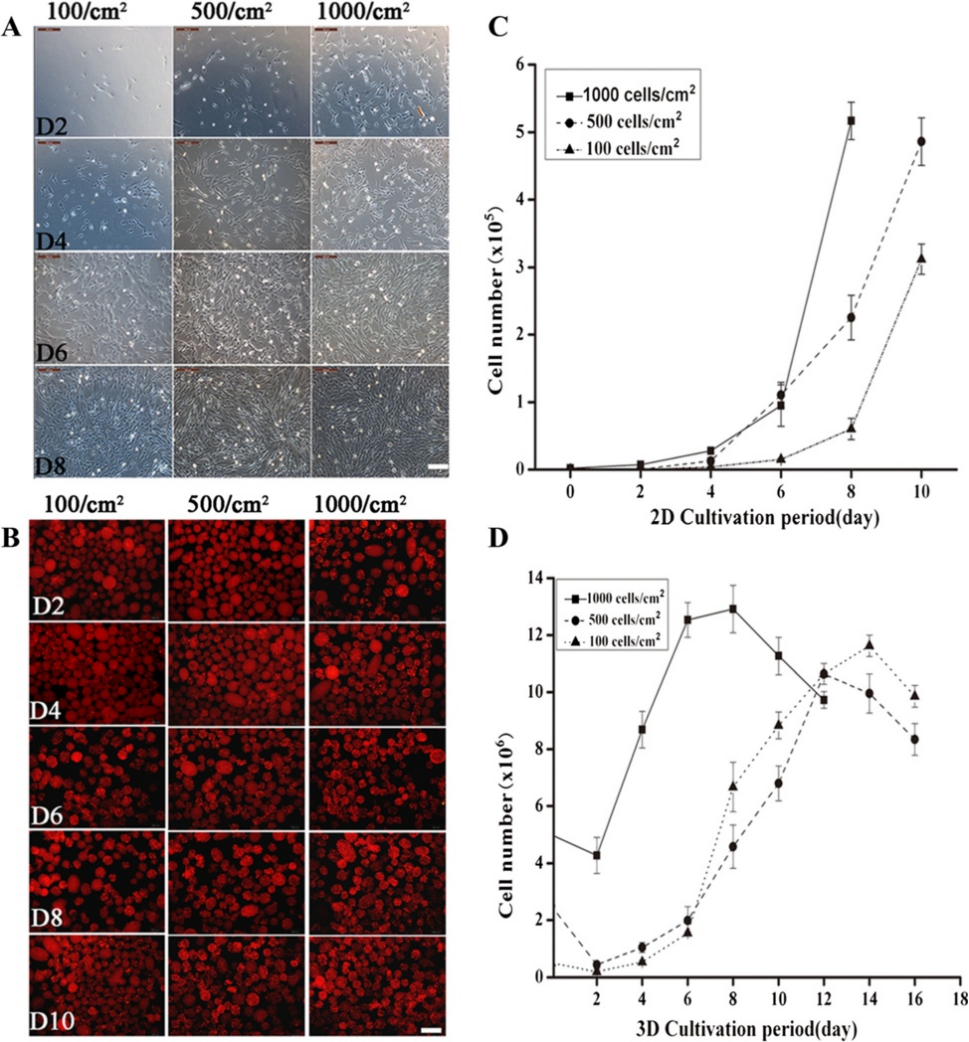
**Proliferation of hWJ-MSC on 2D and 3D cultures.** hWJ-MSC were cultured in tissue culture plates **(A)** or on gelatin microbeads **(B)** for the indicated culture period (six days). hWJ-MSC, seeded at various cell densities, adhered to and proliferated on both tissue culture plates **(C)** or gelatin microbeads, as seen after staining with acridine orange **(D)**, and reached confluence at the indicated time period (day 6 to day 8). 2D, plate cultures; 3D, spinning bottle cultures; hWJ-MSC, human Wharton’s jelly-derived mesenchymal stem cells.

hWJ-MSC, at seeding densities of 100 cells/cm^2^, 500 cells/cm^2^ and 1,000 cells/cm^2^, adhered to and proliferated on tissue culture plates (2D cultures; Figure 2A), and reached confluence at day 8 (100 cells/cm^2^) or day 6 (500 cells/cm^2^, 1,000 cells/cm^2^).

**Figure 3:**
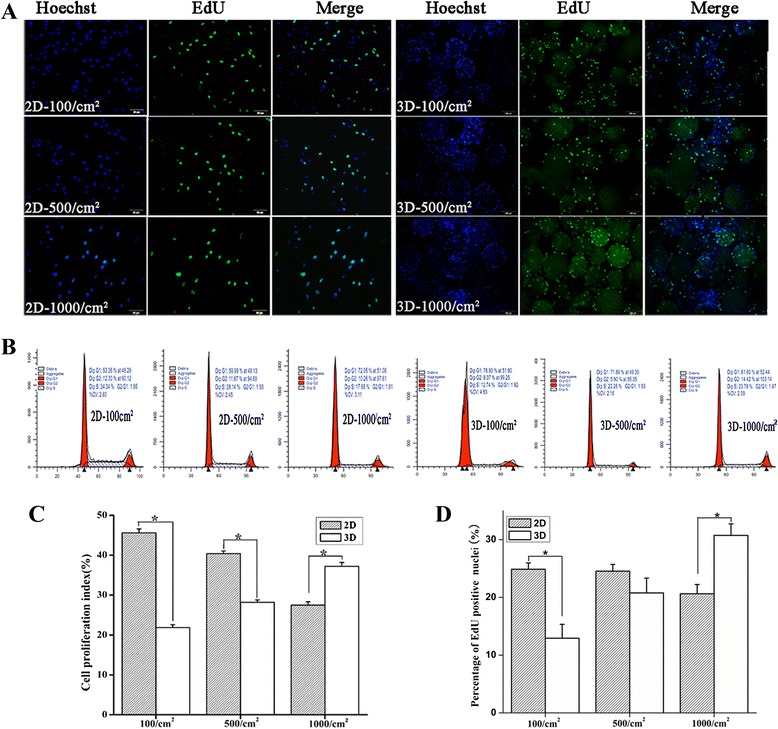
**Cell cycle and EdU assay of hWJ-MSC.** Merged image of EdU staining (green) and Hoechst 33342 staining (blue) for 2D and 3D cultured cells **(A)**, bar = 100 μm. Flow cytometry with propidium iodide staining for 2D cultures and 3D cultures **(B)**. A histogram comparing the percentage of EdU-positive nuclei in 2D and 3D cultured cells.*P <0.05 (2D cultured cells versus 3D cultured cells, at 100 cells/cm^2^), *P <0.05 (3D cultured cells versus 2D cultured cells at 1,000 cells/cm^2^) **(C)**. A histogram **(D)** comparing the cell proliferation index of 2D and 3D cultures. *P <0.05 (2D cultured cells versus 3D cultured cells at 100 cells/cm^2^), *P <0.05 (3D cultured cells versus 2D cultured cells at 1,000 cells/cm^2^). EdU, ethynl-2′-deoxyuridine; hWJ-MSC, human Wharton’s jelly-derived mesenchymal stem cells; 2D, plate cultures; 3D, spinning bottle cultures.

In contrast to 2D cultures, hWJ-MSC cultured on microbeads at these three densities (3D cultures, Figure 2B) entered into the exponential phase as early as two days after commencing 3D culturing; and we found that 1,000 cells/cm^2^ is an optimal seeding density for the 3D culture system, which not only shortens the duration of culture, but also significantly increases cell yields, with as many as 13 million cells generated on 0.5 g gelatin microbeads in a spinning flask (Figure 2C and Figure 2D). EdU incorporation (Figure 3A) and cell cycle assay (Figure 3B) results showed that the cell PI (Figure 3C) and the percentage of EdU-positive (Figure 3D) cells in the 1000-cells/cm^2^ culture density is increased markedly in 3D compared to 2D cultures. Moreover, EdU-positivity and cell PI increased with cell seeding densities in the 3D culture system, but the opposite was true for cells in the 2D culture system.

### SA-β-gal staining and karyotyping analysis of MSCs

**Figure 4:**
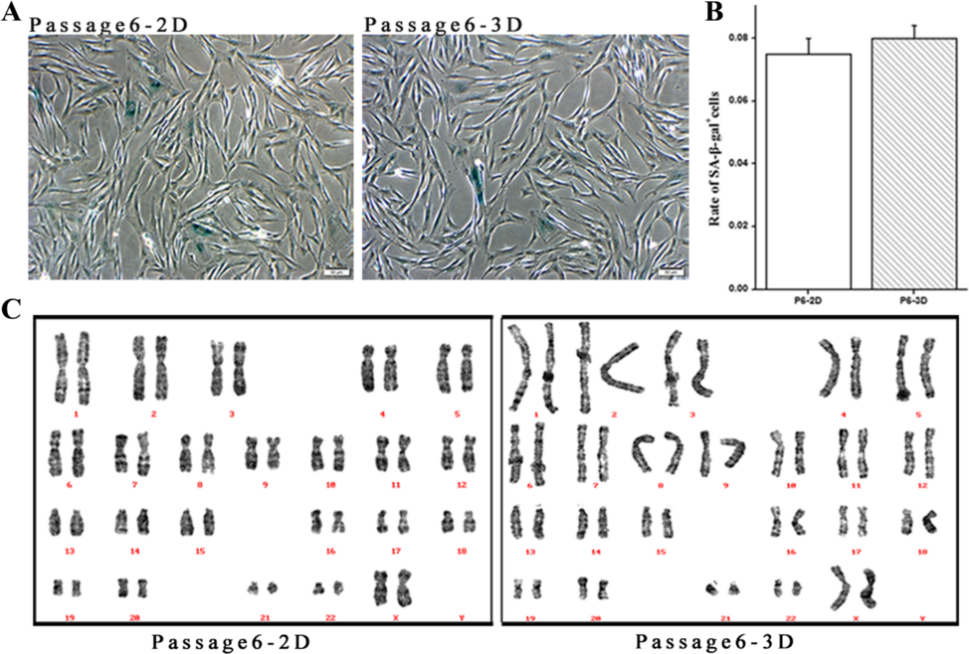
**Senescence and karyotype analysis.** SA-β-gal staining of 2D and 3D cultured hWJ-MSC; blue color represents senescent cells. Scale bar = 50 *μ*m. **(A)** Data were not statistically significantly different between the two groups; cells were counted from three different fields and the average determined. **(B)** Representative karyotypes of cells cultured under 2D and 3D conditions at passage 6. **(C)**. hWJ-MSC, human Wharton’s jelly-derived mesenchymal stem cells; SA-β-gal, senescence-associated β-galactosidase; 2D, plate cultures; 3D, spinning bottle cultures.

To detect the extent of senescence that occurred when the cells were expanded, we conducted SA-β-gal staining. There were no differences between hWJ-MSC cultured in 2D versus 3D culture systems at passage 6 (Figure 4A); the percentage of senescent cells in 3D cultures was 0.075 ± 0.005 and that cells in 2D cultures was 0.08 ± 0.004 (Figure 4B).

To determine the impact of cell culture systems on genomic stability, we performed karyotype analysis, and found that there were no chromosomal abnormalities in hWJ-MSCs cultured in either 2D or 3D systems; at passage 6, a normal female chromosome type (46XX; Figure 4C) was still observed.

### Surface marker expression of hWJ-MSC in 2D and 3D cultures

**Figure 5:**
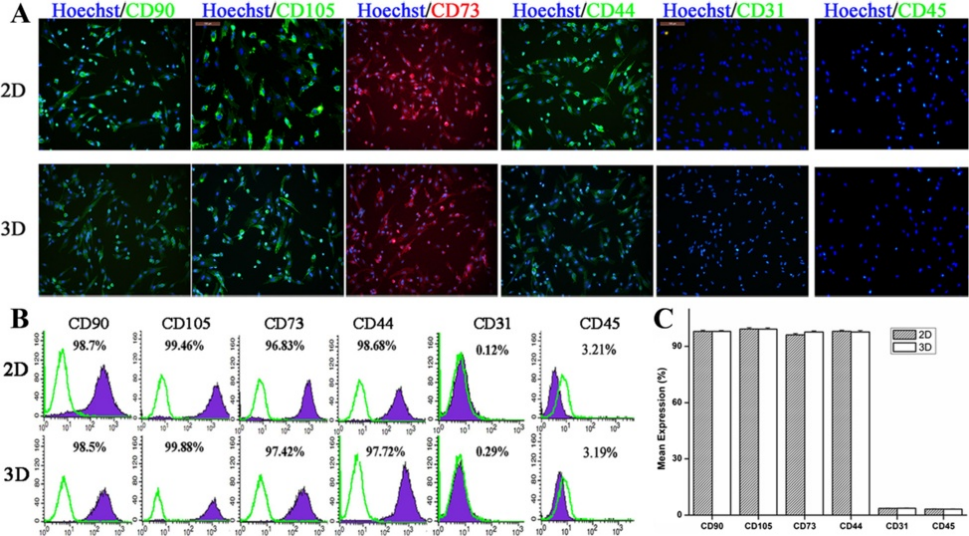
**Surface marker profiles of hWJ-MSC after 2D and 3D culturing.** hWJ-MSC collected from tissue culture plates (2D) or microbeads (3D) were stained with the indicated primary antibodies against CD mesenchymal markers. Alexa Fluor 488- or Alexa Fluor 647-R-phycoerythrin-conjugated secondary antibody was used to detect the primary antibodies. Hoechst 33342 was used to track the nucleus. Immunofluorescence **(A)** and flow cytometry **(B)** results showed that the two types of cultured cells all express the mesenchymal markers CD90, CD105, CD73, and CD44, but did not express CD31 or CD45. Statistical analysis showed no differences in the expression levels of these markers (*P* >0.01) **(C)**. hWJ-MSC, human Wharton’s jelly-derived mesenchymal stem cells.

To determine whether, and to what extent, hWJ-MSC in 3D cultures retained the surface markers of MSCs, immunofluorescence staining and flow cytometry assays were performed on hWJ-MSC derived from 2D and 3D cultures. hWJ-MSC derived from 3D cultures expressed high levels of CD44, CD73, CD90, and CD105, similar to those in hWJ-MSC derived from 2D cultures; neither hWJ-MSC derived from 2D nor those derived from 3D cultures expressed CD31 or CD45 (Figure 5).

### Multipotency of hWJ-MSC derived from 2D and 3D cultures

**Figure 6:**
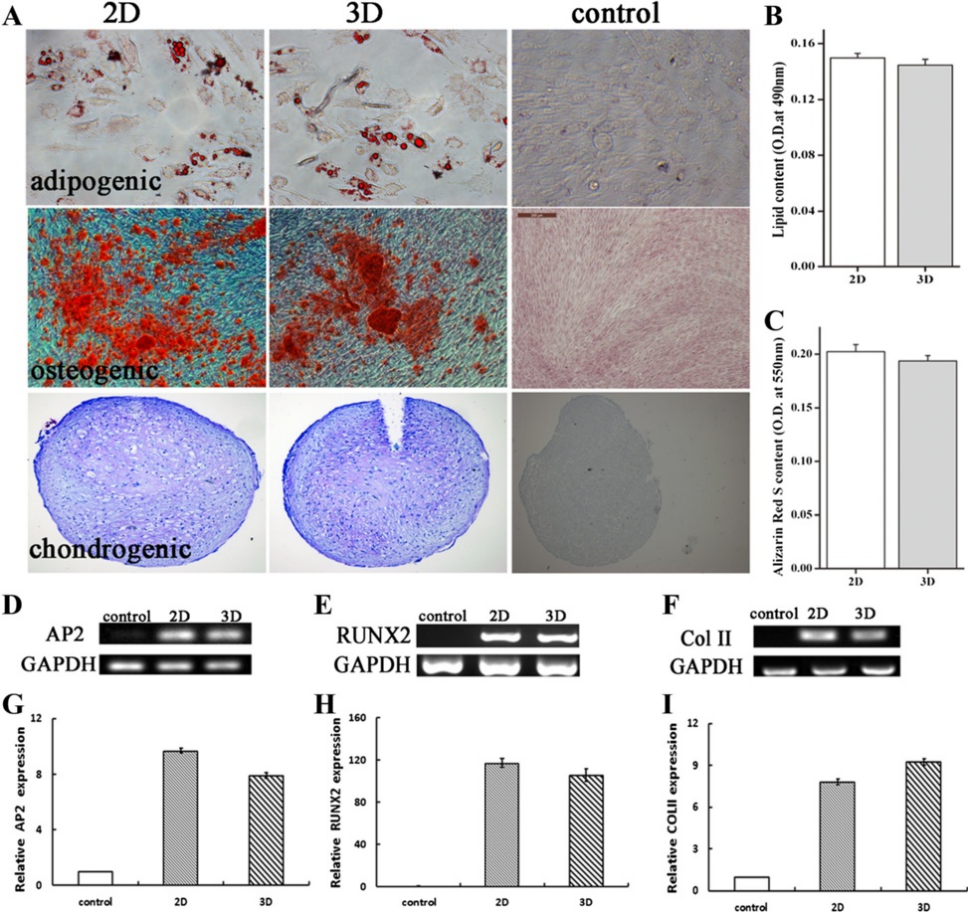
**Multipotency of hWJ-MSC as assessed by chemical staining and qRT-PCR.** hWJ-MSC were collected from tissue culture plates (2D) or microbeads (3D), and cultured in osteogenic, adipogenic, and chondrogenic medium. Oil red O, alizarin red, and toluidine blue were used to evaluate osteogenesis, adipogenesis, and chondrogenesis, respectively; the control groups are shown in **(A)**. A histogram **(B**, **C)** showing the absence of significant differences between 2D cultured cells and 3D cultured cells after staining with oil red O and alizarin red S. RT-PCR showed that the 2D and 3D cultured cells all expressed *AP2* **(D)**, *RUNX2* **(E)**, and *COLII* **(F)**, and the qRT-PCR data indicated that the mRNA levels of *AP2* **(G)**, *RUNX2* **(H)**, and *COLII* **(I)** were not statistically significantly different between 2D cultured cells and 3D culture cells (*P* >0.01). hWJ-MSC, human Wharton’s jelly-derived mesenchymal stem cells.

To determine whether, and to what extent, hWJ-MSC derived from 3D cultures retained multipotency, RT-PCR and immunocytochemistry were performed on hWJ-MSC, derived from 2D and 3D cultures grown under adipogenic, chondrogenic, or osteogenic induction conditions. Oil red O staining, Alizarin red staining, and toluidine blue staining under adipogenic, osteogenic, and chondrogenic induction conditions showed lipid droplet accumulation, calcium nodule formation, and cartilage vacuole formation, respectively, in hWJ-MSC derived from both 2D and 3D cultures (Figure 6A). Moreover, hWJ-MSC derived from 2D and 3D cultures grown under adipogenic and osteogenic induction conditions exhibited a similar degree of adipogenesis and osteogenesis (*P* >0.05; Figure 6B and C).

RT-PCR revealed that hWJ-MSC derived from both 2D and 3D cultures expressed the adipogenic marker *AP2* (Figure 6D), osteogenic marker *RUNX2* (Figure 6E), and chondrogenic marker *COLII* (Figure 6F). Quantitative PCR revealed that the mRNA levels of *AP2* (Figure 6G), *RUNX2* (Figure 6H), and *COLII* (Figure 6I) in hWJ-MSC derived from 3D cultures were very similar to those in cells derived from 2D cultures (*P* >0.05).

### Expression of pluripotent factors in MSCs grown in 2D and 3D culture systems

**Figure 7:**
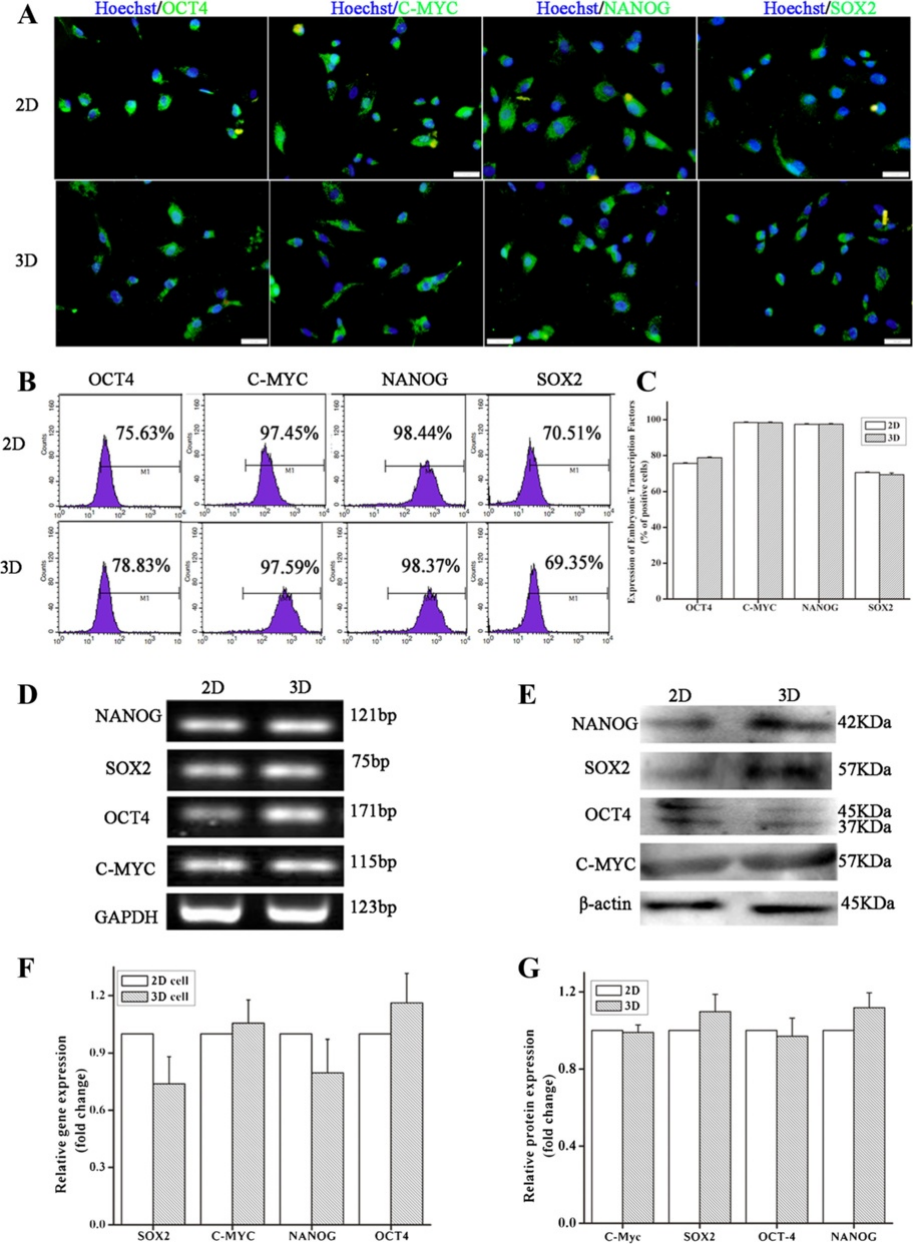
**Expression of pluripotency factors in 2D and 3D cultured cells.** Expression of embryonic markers (green) in 2D and 3D cultured cells, as determined by immunocytochemistry. Cell nuclei were stained with Hoechst 33342 (blue). Scale bar: 20 μm **(A)**. Flow cytometry analysis of C-MYC, OCT4, NANOG, and SOX2 expression in 2D and 3D cultured cells **(B)**. Percentage of cells positive for the expression of embryonic markers as assayed by flow cytometry (n = 4, *P* >0.01) **(C)**. Quantitative real-time polymerase chain reaction assay and Western blot assay for the four factors **(D** to **G**). (n = 4, *P* >0.01). 2D, plate cultures; 3D, spinning bottle cultures.

OCT4, SOX2, NANOG, and C-MYC are core transcription factors that orchestrate regulatory networks and play an important role in maintaining the pluripotency of stem cells. The expression of OCT4, SOX2, NANOG, and C-MYC in hWJ-MSC was determined using immunofluorescence staining, flow cytometry, qRT-PCR, and western blot assays. Immunofluorescence staining (Figure 7A) and flow cytometry analysis (Figure 7B) showed that hWJ-MSC derived from both 2D cultures and from 3D cultures strongly expressed the four core transcription factors, and at similar levels (Figure 7C). These results were supported by PCR and western blot assays results (*P* >0.05; Figure 7D to G).

### Efficacy of hWJ-MSC in enhancing skin wound healing

**Figure 8:**
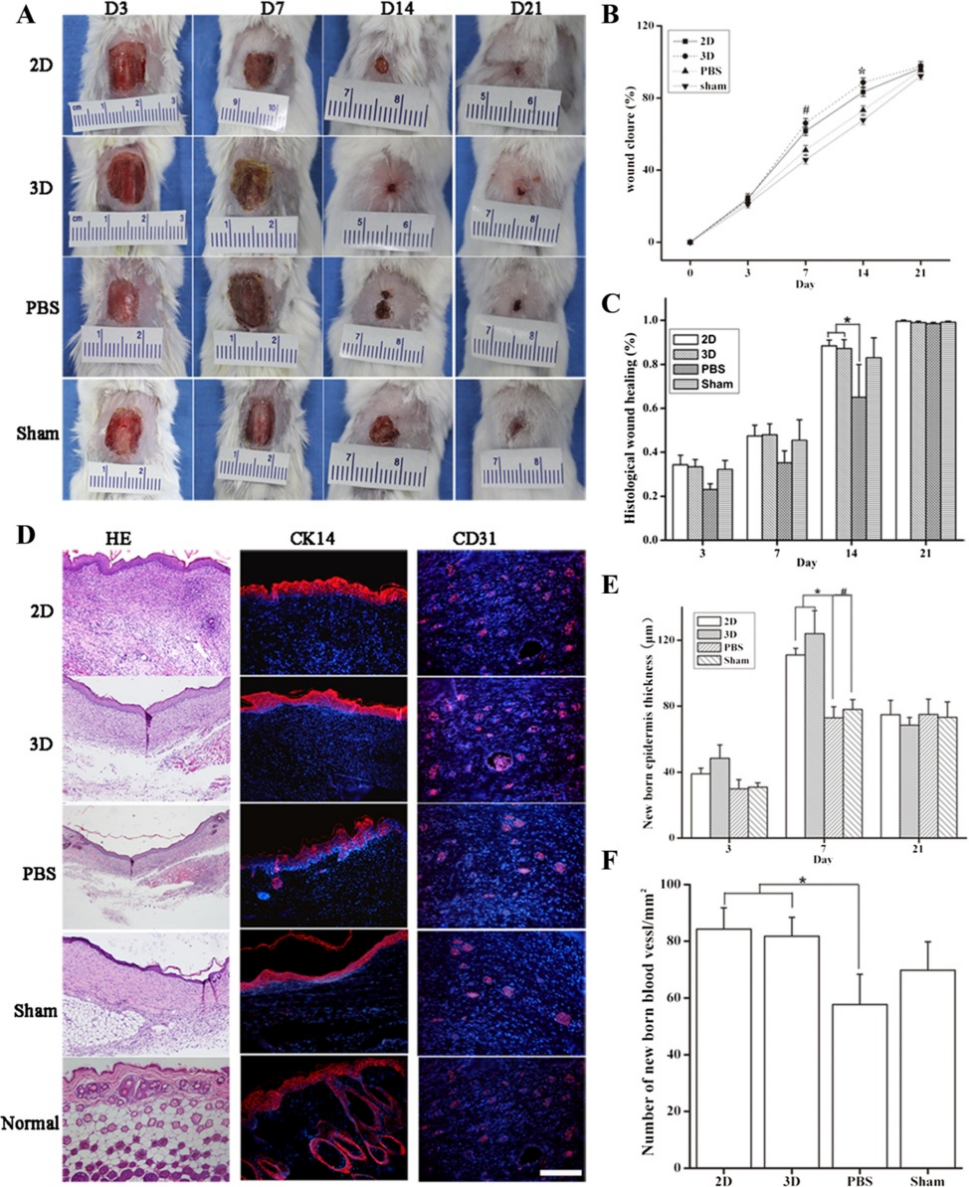
**Efficacy of hWJ-MSC in mouse skin wound closure.** Representative images of the mouse excisional wound splinting model after transplantations of 2D culture-derived cells, 3D culture-derived cells, phosphate-buffered saline (PBS), and control vehicle medium (sham) at days 3, 7, 14, and 21 **(A)**. Wound measurement in each group of mice; n = 6 per group. *P <0.05, for treatment with 2D cultured cells versus PBS, and with 3D cultured cells versus PBS, at day 14. **(B)**. Percentage of histological wound healing; n = 6 per group, for treatment with 2D cultured cells versus PBS; *P <0.05; or for 3D cultured cells versus PBS; *P <0.05; or for 2D cultured cells versus 3D cultured cells; *P* >0.05, at day 14 **(C)**. Wound histology after hematoxylin and eosin staining. Tissue sections obtained from the wound area at day 14 after cell injection were stained with antibodies against cytokeratin 14 (red) and CD31 (red). Fluorescence microscopy showed that cytokeratin (red) was expressed in the newly formed epidermis and that CD31 (red) was present in the newly formed blood vessels **(D)**. The thickness of the new epidermis was measured using Image J. There was no significant difference between groups at days 7 and 21 after transplantation. The thickness of the newly formed epidermis in animals treated with 2D and 3D cultured cells were both greater than that seen in the PBS- and sham-treated groups **(E)**. A histogram representing the number of newly formed blood vessels in 1 mm^2^ by day 14 after transplantation. 2D versus 3D cultured cells, *P* >0.01; 2D cultured cells versus PBS, *P* <0.05, 3D cultured cells versus PBS, *P* <0.05; 2D cultured cells versus sham, *P* >0.05, 3D cultured cells versus sham, *P* >0.05 **(F)**. hWJ-MSC, human Wharton’s jelly-derived mesenchymal stem cells; 2D, plate cultures; 3D, spinning bottle cultures.

To investigate the efficacy of hWJ-MSC in skin wound healing, hWJ-MSC derived from 2D and 3D cultures were implanted into full-thickness skin wounds. Gross inspection showed that treatment with hWJ-MSC derived from either 2D or 3D cultures enhanced wound healing by day 14, as compared with PBS treatment (61.66% ± 2.6% (2D) versus 66.02% ± 2.6% (3D); *P* >0.05; 61.66% ± 2.6% (2D) versus 45.67% ± 2.3% (PBS); *P* <0.05; 66.02% ± 2.6% (3D) versus 45.67% ± 2.3% (PBS); *P* <0.05; Figure 8A and B).

H & E staining showed that full-thickness defects of skin implanted with hWJ-MSC derived from 2D or 3D cell cultures exhibited significantly more re-epithelialization than those treated with PBS on day 14 (88% ± 0.04% (2D) versus 65% ± 0.14% (PBS), *P* <0.05; 87% ± 0.02% (3D) versus 65% ± 0.14% (PBS), *P* <0.05; 88% ± 0.04% (2D) versus 87% ± 0.02% (3D), *P* >0.05; Figure 8C and D).

### Engraftment potential of 2D versus 3D culture-derived cells

Histological analysis and cytokeratin 14 staining showed that the thickness of the newly formed epidermis in the wounded skin that had been implanted with hWJ-MSC was significantly thicker than that in the PBS-treated group and the sham group at day 14 (111.01 ± 4.07 μm (2D) versus 72.84 ± 6.78 μm (PBS), *P* <0.05; 123.94 ± 4.02 μm (3D) versus 72.84 ± 6.78 μm (PBS), *P* <0.05; 111.01 ± 4.07 μm (2D) versus 78.00 ± 5.93 μm (sham), *P* <0.05; 123.94 ± 4.02 μm (3D versus 78.00 ± 5.93 (sham), *P* <0.05; 111.01 ± 4.07 μm (2D) versus 123.94 ± 4.02 μm (3D), *P* >0.05; Figure 8D and E).

To detect angiogenesis in the skin wound, we performed immunofluorescence staining using anti-CD31 antibody to detect endothelial cells in the lumen of newly formed blood vessels. Our immunofluorescence staining showed that the densities of the newly formed capillary vessels in the skin wounds implanted with hWJ-MSC derived from 2D or 3D cultures were significantly higher than those in the wounds treated with PBS at day 14 (84.25 ± 7.57 (2D) versus 57.75 ± 7.82 (PBS), *P* <0.05; 81.75 ± 6.65 (3D) versus 57.75 ± 7.82 (PBS), *P* <0.05; 84.25 ± 7.57 (2D) versus 81.75 ± 6.65 (3D), *P* >0.05; Figure 8D and F).

## Discussion

In this study, we developed a 3D cell culture system for generating high yields of hWJ-MSC. In this 3D culture system, the cells not only demonstrated genomic stability and reduced senescence, but also retained the surface markers of MSCs, multipotency differentiation ability, a high proliferation potential, and efficacy in enhancing skin wound healing and angiogenesis that was comparable to that of hWJ-MSC derived from 2D cultures.

Generation of stem cells on a large scale, while maintaining the cells in a highly proliferative and multipotent state, is a major aim in the field of stem cell therapy. The use of microbeads as carriers for cell cultures circumvents the shortcomings related to 2D culture, in which the expansion of stem cells is costly, time-consuming, and labor-intensive [37]. The chemical composition, surface topography, degree of porosity, and the charge density of the microbeads, and the resulting surface area, determine the success of such stem cell cultures [33-36].

Being anchorage dependent cells, MSCs need to be attached to a surface for expansion. Microbeads have a large surface/volume ratio, are non-toxic to cells, and offering superb culture surfaces for adhesion and proliferation of anchorage-dependent cells, within a limited culture space. CultiSpher-G macroporous gelatin microbeads are the most commonly used scaffold materials and have been successfully used in a wide range of applications [47]. These microbeads are composed of collagen, and are biocompatible and can be degraded in the human body [38], whereas other microcarrier materials, such as glass microcarriers and Cytodex-3 microbeads, cannot be digested in the body [34]. This biodegradable characteristic allows these gelatin beads to be used as vehicles to deliver cultured cells directly to the tissues that require regeneration, without having to release the cells from culture surfaces by trypsinization before the initiation of cell therapy, which decreases the yield of cells [39,40].

Collagen is one of the main components of the extracellular matrix; it synergistically and coordinately interacts with other extracellular matrix components in the body, offering an optimal niche to stem cells for their self-renewal and commitment to differentiation into tissue-specific cells. Thus, collagen-derived gelatin microbeads not only supply a superb surface area for cell adhesion and proliferation [32], but also mimic the *in vivo* environment more closely than do other materials; this potentially contributes to expansion of stem cells on a large scale and maintenance of the stem cells in a multipotent state.

The use of this material has recently been reported in a variety of applications, including the culturing of human chondrocytes and the chondroprogenitor cells, but the feasibility of using Cultispher-G microcarriers for the expansion of hWJ-MSC in a stirred suspension culture system had not been reported to date. Moreover, cells cannot attach firmly to microcarriers made of other components because of the smooth surface and thereby retain a rounded morphology on the microcarriers during culturing. Because of this fragile bonding, cells might detach and be washed away during medium changes. The microcarrier beads we used have large apertures that offer cells adequate space for proliferation, and the cells can easily attach firmly to these surfaces even while being stirred in the 3D culture system [48]. In our study, there was a significant increase in the number of cells cultured on the microcarriers as compared to those cultured in monolayer.

For microcarrier culture systems, use of an optimal inoculating density is crucial [49]. In our study, we chose three different seeding densities; we found that a higher density (1,000 cells/cm^2^) was better than a lower cell density (100 cells/cm^2^, 500 cells/cm^2^). For the 3D culturing systems, using a density of 1,000 cells/cm^2^, expansion of cells started with a two-day lag-phase, then entered the exponential growth phase, and the number of cells increased maximally by 2.5-fold. In the future, we plan to investigate whether the rotation speed of the bioreactor affects the proliferation of cells.

Most studies on the use of microcarriers for the expansion of MSCs have not reported whether, and to what extent, the cells retain MSC characteristics [34,48,49], even though that is the key requirement for stem cell expansion for use in clinical therapy. We determined the expression of surface markers, multilineage differentiation, and self-renewal capacity of the cells derived by 3D culturing, using qualitative and quantitative analysis. There were no significant differences in the stem cell characteristics of cells cultured in 2D and 3D systems; cells derived by both cell culture systems expressed the surface markers CD90, CD105, CD73, and CD44, but did not express CD31 or CD45; and multipotency analysis showed that the 3D culture-derived cells have the ability to differentiate into adipogenic, osteogenic, and chondrogenic lineages. In previous similar studies, pluripotency analysis showed that chondroprogenitor cells, mouse embryonic stem cells, and human embryonic stem cells could be produced after expansion of stem cells on microcarriers, but most of these were detected by staining. In order to verify the multiple differentiation potential of the cells, we focused specifically on the cellular and gene expressions level of the microcarrier-expanded cells. We found that the 3D culture-derived cells still expressed the adipogenesis gene *AP2*, the osteogenesis gene *RUNX2*, and the chondroprogenitor gene *COLII*. In future, we will focus on others genes to further investigate whether there are differences between 2D and 3D culture-derived cells.

OCT4, SOX2, NANOG, and C-MYC are considered core transcription factors. They interact with each other to form key regulatory networks and subsequently maintain stem cells in a self-renewal and pluripotent state. These factors are also found in stromal cells, such as bone marrow (BM)-derived MSCs and umbilical cord-derived MSCs, but gradually disappear with repeated subculturing of stem cells. To quantify the expression of the core transcription factors in hWJ-MSC derived from 3D cultures, we used real-time PCR, western blotting, and flow cytometry and found that the expression of OCT4, SOX2, NANOG, and C-MYC in hWJ-MSC derived from 3D cultures was similar to that in cells derived from 2D cultures, suggesting that hWJ-MSC expanded in 3D cultures retained a multipotency similar to those expanded in 2D cultures. Determining the extent to which cells cultured on microcarriers express the four transcription factors lays the basis for further investigation of expanding induced pluripotent stem cells (iPS) in 3D culture systems.

Chronic wounds are common conditions that are difficult to heal, involving complex, orchestrated processes, which include the phases of inflammation, angiogenesis, formation of granulation tissue, re-epithelialization, and fibro-proliferation or matrix formation. hWJ-MSC can enhance wound healing via paracrine signaling [50,51]. It has been suggested that MSCs can secrete many different cytokines and growth factors to influence cell migration and proliferation *in vitro* and *in vivo* [52,53]. Because harvesting of hWJ-MSC is not painful or invasive, unlike that of bone marrow derived-meschymal stem cells (BM-MSCs) and adipose tissue-derived stromal cells (ADSCs), and because Wharton’s jelly is readily available, is a rich source of MSCs, and represents an immune-privileged tissue, hWJ-MSC is a particularly attractive stem cell source with potential for application in cell-based regenerative medicine. Here, to test the efficacy of Bio-MCCS-cultured hWJ-MSC in skin wound healing, hWJ-MSC were implanted into full-thickness excisional skin wounds on the dorsal surface of mice. Gross inspection showed that hWJ-MSC derived from either 2D or 3D cultures exhibited similar efficacies in enhancing skin wound healing, which was significantly better than in the control or sham groups. Indeed, the resurfacing of recalcitrant chronic skin leg ulcers and regimentation of vitiligo by direct administration of keratinocytes or melanocytes cultured on gelatin microbeads has been very successful.

## Conclusions

In summary, we generated hWJ-MSC on a large scale using Cultispher-G microcarriers in stirred spinner bottles. Furthermore, from detailed qualitative and quantitative analysis, our data indicated that the hWJ-MSC expanded in our 3D culture system have not undergone genomic change and have retained the surface markers of MSCs and the expression of core transcription factors, self-renewal, and multipotent differentiation potential, as well as biological efficacy in enhancing skin wound healing, in a manner comparable to that of hWJ-MSC derived by 2D culture. Our study showed that hWJ-MSC can be successfully expanded on a large scale using our stirred microcarrier culture system, while maintaining their stem cell properties, which can offer rich stem cell sources for cell biology studies and clinical applications.

## Abbreviations

2D: plate cultures
3D: spinning bottles cultures
ADSCs: adipose tissue-derived stromal cells
ANOVA: analysis of variance
bFGF: basic fibroblast growth factor
BM-MSC: bone marrow-derived mesenchymal stem cells
bp: base pair
BSA: bovine serum albumin
DAPI: 4′,6-diamidino-2-phenylindole
(D)MEM/F12: (Dulbecco’s) modified Eagle medium/F12
EdU: 5-ethynyl-2′-deoxyuridine
FBS: fetal bovine serum
FITC: fluorescein isothiocyanate
H & E: hematoxylin and eosin
HRP: horseradish peroxidase
hWJ-MSC: human Wharton’s jelly-derived mesenchymal stem cells
MSCs: mesenchymal stem cells
OD: optical density
PBS: phosphate-buffered saline
PBST: phosphate-buffered saline/TweenX20 or TritonX100
PCR: polymerase chain reaction
PI: proliferation index
RT -PCR: real time-polymerase chain reaction
SA-β-gal: senescence-associated β-galactosidase
SD: standard deviation
